# Annexin A1 drives macrophage skewing to accelerate muscle regeneration through AMPK activation

**DOI:** 10.1172/JCI124635

**Published:** 2020-02-04

**Authors:** Simon McArthur, Gaëtan Juban, Thomas Gobbetti, Thibaut Desgeorges, Marine Theret, Julien Gondin, Juliana E. Toller-Kawahisa, Chris P. Reutelingsperger, Bénédicte Chazaud, Mauro Perretti, Rémi Mounier

**Affiliations:** 1 Institute of Dentistry and; 2 William Harvey Research Institute, Barts and the London School of Medicine and Dentistry, Queen Mary University of London, London, United Kingdom.; 3 Université Claude Bernard Lyon 1, CNRS UMR-5310, INSERM U-1217, Institut NeuroMyoGène, Lyon, France.; 4 Department of Biochemistry and Immunology, Ribeirão Preto Medical School, University of São Paulo, São Paulo, Brazil.; 5 Department of Biochemistry and; 6 Cardiovascular Research Institute Maastricht, Faculty of Health, Medicine and Life Sciences, Maastricht University, Maastricht, Netherlands.; 7 Centre for Inflammation and Therapeutic Innovation, Queen Mary University of London, London, United Kingdom.

**Keywords:** Inflammation, Muscle Biology, Homeostasis, Macrophages, Protein kinases

## Abstract

Understanding the circuits that promote an efficient resolution of inflammation is crucial to deciphering the molecular and cellular processes required to promote tissue repair. Macrophages play a central role in the regulation of inflammation, resolution, and repair/regeneration. Using a model of skeletal muscle injury and repair, herein we identified annexin A1 (AnxA1) as the extracellular trigger of macrophage skewing toward a pro-reparative phenotype. Brought into the injured tissue initially by migrated neutrophils, and then overexpressed in infiltrating macrophages, AnxA1 activated FPR2/ALX receptors and the downstream AMPK signaling cascade, leading to macrophage skewing, dampening of inflammation, and regeneration of muscle fibers. Mice lacking AnxA1 in all cells or only in myeloid cells displayed a defect in this reparative process. In vitro experiments recapitulated these properties, with AMPK-null macrophages lacking AnxA1-mediated polarization. Collectively, these data identified the AnxA1/FPR2/AMPK axis as an important pathway in skeletal muscle injury regeneration.

## Introduction

An efficient inflammatory response is a necessary component of the reaction to injury or infection, but it is equally critical for this inflammatory response to be terminated in a timely and appropriate manner, enabling the restoration of tissue homeostasis (1, 2). Indeed, chronic inflammation that results from failure of resolution represents a major contributing factor to a multitude of pathologies, from arthritis to sepsis to dementia (3, 4).

An inflammatory reaction consists of the coordinated activity of numerous cells and soluble mediators, but a central role is played by the macrophage. These cells, whether resident in the tissue or recruited from circulating monocyte populations, are among the first responders to pathogen- or damage-associated molecular patterns, initiating endothelial activation and neutrophil recruitment (5). Beyond their roles as sentinels, macrophages are important drivers of the progression of an inflammatory response, acting to clear pathogens, effete cells, and debris by phagocytosis (6), serving as antigen-presenting cells to recruit the adaptive arm of the immune response (7), and ultimately enabling processes of tissue repair and resolution (8, 9). That one cell type is able to achieve this diverse array of functions is due in large part to its remarkable degree of phenotypic plasticity, and, as such, macrophages may exist in a wide variety of forms along a spectrum running from a largely proinflammatory state, often indicated as M1, to a primarily nonphlogistic and pro-resolving phenotype, termed M2 (10).

A number of factors promoting this phenotypic transformation have been studied, including exposure to antiinflammatory cytokines such as IL-10 and IL-4, and the phagocytic removal of cell debris, although a complete description of the underlying mechanisms is lacking (11). We and others have identified a key role for the intracellular signaling pathway governed by AMP-activated protein kinase (AMPK) (12–14). Activation of this pathway is required for efficient conversion of pro- to antiinflammatory-type macrophages, and inhibition of such a response significantly attenuated recovery in a model of inflammatory skeletal muscle injury (13). While AMPK is undoubtedly important in the phenotypic conversion of macrophages during the course of an inflammatory reaction, the nature of the extracellular trigger(s) for its stimulation remains unclear.

The protein annexin A1 (ANXA1) (15, 16) is a major driver of inflammatory resolution, promoting neutrophil apoptosis (17), nonphlogistic monocyte recruitment (18), and macrophage efferocytosis (19, 20). Moreover, we and others have recently provided evidence showing ANXA1 to promote an antiinflammatory macrophage phenotype in in vitro models of rheumatoid arthritis (21) and tumor growth (22), but how this translates to an in vivo situation is less clear. Against this background, we applied a well-characterized model of skeletal muscle injury (23–25) to test the hypothesis that ANXA1 and its receptor FPR2/ALX could be the upstream trigger of AMPK activation, and hence be a major driver of the pro- to antiinflammatory macrophage phenotype shift, promoting inflammatory resolution and the restoration of skeletal muscle tissue homeostasis.

## Results

### AnxA1- and Fpr2/3-null mice show impaired recovery from skeletal muscle injury.

To investigate the role of ANXA1 and its receptor FPR2/ALX (in humans, or the ortholog Fpr2/3 in mice) in the control of macrophage phenotype, we first used the murine model of cardiotoxin-induced tibialis anterior (TA) injury, a model characterized by necrotic tissue damage and extensive macrophage activity (13, 26).

**Figure 1:**
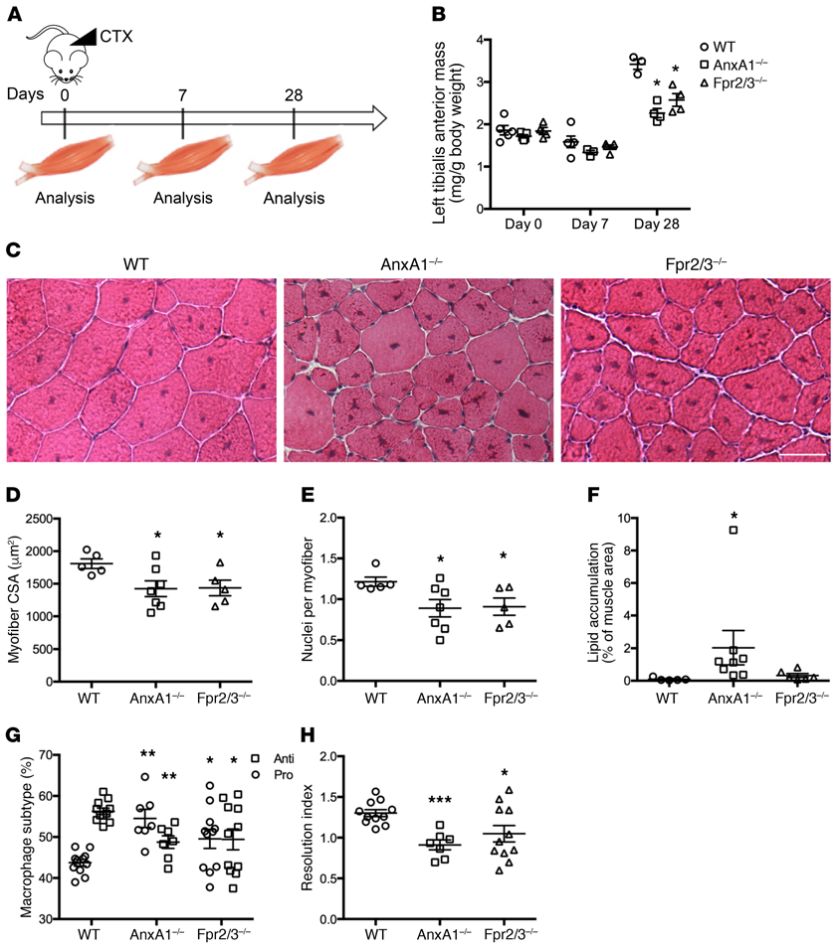
Nonredundant role of ANXA1 in cardiotoxin-induced muscle injury and repair. (**A**) Experimental setup. Acute injury was induced by cardiotoxin (CTX) injection in the tibialis anterior (TA) of WT, AnxA1^–/–^, and Fpr2/3^–/–^ mice. Muscles were analyzed 0, 7, and 28 days after injury. (**B**) TA mass normalized to mouse body weight. (**C**) H&E staining of muscles 28 days after injury. Scale bar: 50 μm. Myofiber cross-sectional area (**D**), number of nuclei per myofiber (**E**), and lipid accumulation (**F**) in muscles 28 days after CTX injury. (**G** and **H**) Macrophage subtype analysis 2 days after CTX injury. Shown are the percentage of pro- and antiinflammatory macrophages within the F4/80^+^ population (**G**) and the resolution index (**H**). Results are mean ± SEM of at least 3 animals. *P < 0.05, **P < 0.01, ***P < 0.001 vs. WT.

While uninjured TA muscle weight and myofiber size were comparable between WT, AnxA1^–/–^, and Fpr2/3^–/–^ mice (Figure 1, A and B, and Supplemental Figure 1, A and B; supplemental material available online with this article; https://doi.org/10.1172/JCI124635DS1), recovery of muscle mass following administration of cardiotoxin was significantly reduced in AnxA1^–/–^ and Fpr2/3^–/–^ mice compared with WT animals (Figure 1B). Histological analysis of muscles 28 days after injury revealed significantly reduced myofiber cross-sectional area and myonuclei per fiber (i.e., the result of differentiation and fusion of muscle stem cells) in AnxA1^–/–^ and Fpr2/3^–/–^ animals (Figure 1, C–E , and Supplemental Figure 1). These outcomes are suggestive of impaired skeletal muscle regeneration, a notion confirmed by marked lipid accumulation in AnxA1^–/–^ mice (Figure 1F and Supplemental Figure 1D). Analysis of immune cell infiltration by flow cytometry revealed that 2 days after cardiotoxin administration, approximately 60% of macrophages expressed a pro-resolving, antiinflammatory phenotype (CD45^+^Ly6C/G^neg^F4/80^hi^) in WT mice, while AnxA1^–/–^ and Fpr2/3^–/–^ animals showed comparable levels of proinflammatory (CD45^+^Ly6C/G^hi^F4/80^lo^) and antiinflammatory (CD45^+^Ly6C/G^lo^F4/80^hi^) cells (Figure 1G and Supplemental Figure 1E). Consequently, the resolution index (ratio of anti- to proinflammatory macrophages) of AnxA1^–/–^ and Fpr2/3^–/–^ mice was significantly lower than that of WT animals, indicative of a prolonged inflammatory response (Figure 1H). These results indicate that a proportion of proinflammatory macrophages failed to convert to an antiinflammatory phenotype at the time of resolution in the absence of AnxA1 or Fpr2/3. Inactivation of the Fpr1 receptor, another member of the Fpr family, did not reproduce these results (Supplemental Figure 1, F and G), suggesting a specific role of the AnxA1-Fpr2/3 pathway in these settings of cardiotoxin-induced muscle injury and repair.

### Macrophages are the major source of AnxA1 in lesion recovery.

**Figure 2:**
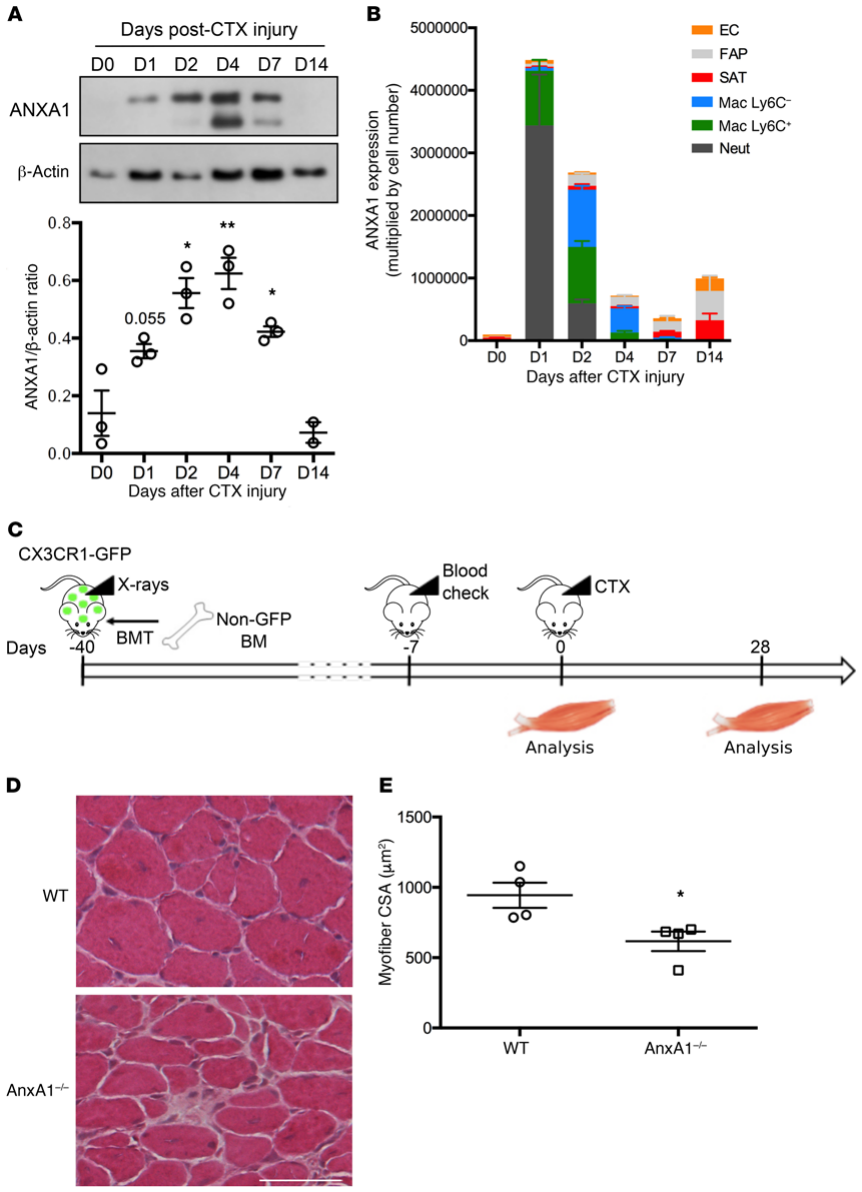
Infiltrating myeloid cell–derived ANXA1 controls muscle repair. (**A**) Western blot analysis of ANXA1 protein in total TA muscle. Muscles were analyzed 0, 1, 2, 4, 7, and 14 days after injury. Shown are representative blots (top) and quantification of ANXA1 to β-actin (bottom) and ratios. (**B**) Quantitative reverse transcriptase PCR analysis of AnxA1 mRNA level in various cell populations FACS-sorted from TA muscle. Muscles were analyzed 0, 1, 2, 4, 7, and 14 days after injury. EC, endothelial cells; FAP, fibro/adipogenic progenitors; SAT, satellite cells; Mac, macrophages; Neut, neutrophils. (**C**) Experimental setup of bone marrow transplantation (BMT). CX3CR1-GFP mice were irradiated and then transplanted with bone marrow cells isolated from WT or AnxA1^–/–^ mice. Bone marrow engraftment was checked on a blood sample after around 5 weeks. Then animals were injured in their TA by CTX injection and muscles analyzed 0 or 28 days later. Engraftment was confirmed on the bone marrow of each animal on the day of sacrifice. H&E staining (**D**) and myofiber cross-sectional area (**E**) of TA muscles 28 days after CTX injury. Scale bar: 50 μm. Results are mean ± SEM of at least 2 (D14 in **A**) or 3 muscles. *P < 0.05, **P < 0.01 vs. WT or D0.

The more pronounced effects of cardiotoxin in AnxA1^–/–^ and Fpr2/3^–/–^ mice indicate that this pathway exerts important regulatory functions in skeletal muscle injury. We then characterized AnxA1 and Fpr2/3 expression kinetics during skeletal muscle regeneration. Monitoring ANXA1 expression in the tissue revealed that, while essentially undetectable in uninjured tissue, the protein was transiently induced from day 1 to day 4 after lesioning, with much lower levels by day 7 (Figure 2A). AnxA1 mRNA analysis on FACS-sorted cell populations (Figure 2B and Supplemental Figures 2 and 3A) combined with immunostaining (Supplemental Figure 3, B–D) indicated that this mediator was primarily restricted to immune cells until day 2 after lesioning. While F4/80^+^ murine macrophages expressed AnxA1 at all time points examined (Supplemental Figure 3, B and C), the proportion of macrophages expressing its primary receptor, Fpr2/3, decreased over time, from approximately 95% at day 2 to 70% at day 7, and finally to 5% 2 weeks after lesioning, even though significant numbers of macrophages were still detected in the tissue (Supplemental Figure 4, B and C). Importantly, expression of Fpr2/3 on muscle fibers was not apparent at any time point examined, as it was restricted to neutrophils and proinflammatory macrophages (Supplemental Figure 4).

We next sought to functionally validate the role of this immune cell–derived ANXA1 using chimeric mice bearing WT muscle but AnxA1^–/–^ bone marrow–derived leukocytes. Therefore, CX3CR1-GFP mice, which harbor GFP-expressing monocytes/macrophages, were irradiated and transplanted with WT- or AnxA1^–/–^-derived bone marrow cells, before injection of cardiotoxin in the TA muscle (Figure 2C). Analysis of bone marrow populations at the time of euthanasia showed that less than 1% of monocytes (CD115^+^ cells) expressed GFP, suggesting greater than 99% engraftment efficiency with either WT or AnxA1^–/–^ bone marrow (Supplemental Figure 5, B and C). Weight recovery was similar after WT or AnxA1^–/–^ transplant (Supplemental Figure 5A), but histological analysis of TA muscles 28 days after cardiotoxin injury revealed that animals receiving AnxA1^–/–^ bone marrow cells displayed a significantly reduced myofiber cross-sectional area compared with animals receiving WT bone marrow cells (Figure 2, D and E). Since more than 90% of the macrophages present in the injured muscles originated from the transplanted marrow (Supplemental Figure 5, D and E), these data indicate that the defective regeneration quantified in AnxA1^–/–^ muscle is a consequence of an intrinsic defect in myeloid, rather than stromal, cells.

### Exogenous ANXA1 can induce human macrophage phenotype conversion in vitro.

**Figure 3:**
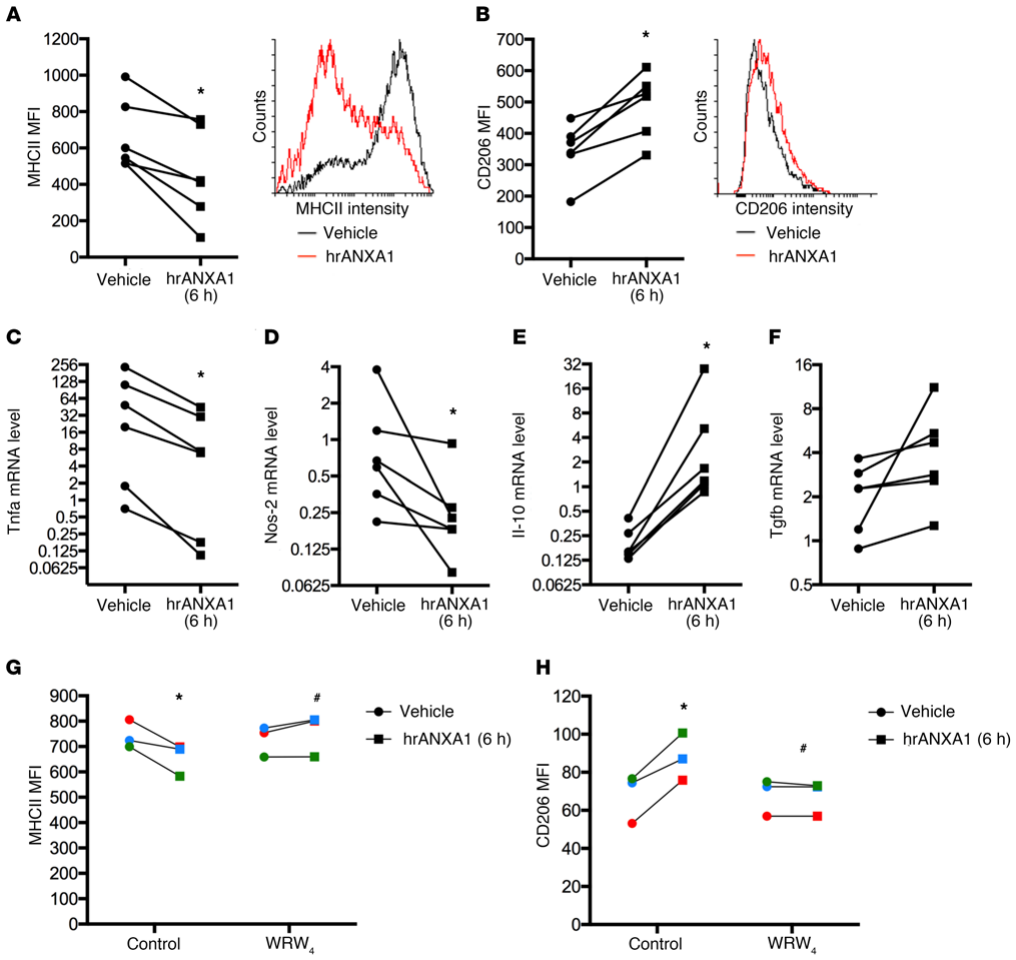
Exogenous hrANXA1 controls human and mouse macrophage polarization in vitro. Human PBMC–derived macrophages were incubated for 24 hours with lipopolysaccharide plus IFN-γ to promote an M1-like phenotype, before addition of human recombinant ANXA1 (hrANXA1; 10 nM) for a further 6 hours. (**A** and **B**) Median fluorescence intensity (MFI) units measured by flow cytometry of MHCII proinflammatory (**A**) and CD206 antiinflammatory (**B**) markers. Shown are MFI quantification (left) and representative FACS plots (right). (**C**–**F**) Quantitative reverse transcriptase PCR analysis of Tnfa (**C**) and Nos-2 (**D**) proinflammatory genes, and Il-10 (**E**) and Tgfb1 (**F**) antiinflammatory genes. (**G** and **H**) MFI units as measured by flow cytometry of MHCII proinflammatory (**G**) and CD206 antiinflammatory (**H**) markers after treatment by hrANXA1 in the presence or absence of the FPR2/ALX antagonist WRW_4_ (10 μM). Experiments were performed on PBMCs from 6 (**A**–**F**) or 3 (**G** and **H**) independent donors. Each color represents an independent PBMC donor. *P < 0.05 vs. vehicle; ^#^*P* < 0.05 vs. control.

As our murine analyses indicate a potential role for AnxA1 in the polarization of macrophages from a proinflammatory to a pro-resolving/reparative phenotype, we investigated these effects in vitro using human PBMC–derived macrophages. An M1-like macrophage phenotype was induced by 24 hours of incubation with bacterial lipopolysaccharide and IFN-γ, prior to addition of human recombinant ANXA1 (hrANXA1). Following hrANXA1 treatment, analysis of cell surface markers revealed a significant reduction in expression of the M1 marker protein major histocompatibility complex II (MHCII) (Figure 3A), accompanied by a significant increase in expression of the M2 marker protein CD206 (Figure 3B). These surface marker changes were mirrored by changes at the transcriptional level, with hrANXA1 treatment inducing a reduction in mRNA expression for the proinflammatory genes *Tnfa* and *Nos2* paired with increased message of *Il-10* (Figure 3, C–E). No significant changes in *Tgfb1* expression were quantified (Figure 3F). Together, these in vitro data support our in vivo findings, providing evidence that ANXA1 application favors a pro-resolving macrophage phenotype.

Intriguingly, we observed significant FPR2/ALX surface expression in both unstimulated human PBMC–derived macrophages and M1-phenotype cells, yet cell surface expression of the receptor was lost on cells stimulated toward an M2 phenotype with IL-4 (Supplemental Figure 6). These data are in agreement with the in vivo observation that Fpr2/3^–/–^ is absent from macrophages that have infiltrated the injured muscle at day 7 and beyond, that is, when a reparative cell phenotype has been acquired (Supplemental Figure 4). The functional engagement of FPR2/ALX was then confirmed through the use of the selective antagonist WRW_4_. The modulatory effects of hrANXA1 on proinflammatory PBMC-derived macrophages (i.e., reduced surface MHCII expression and augmented surface CD206 expression) were lost in the presence of WRW_4_ (Figure 3, G and H).

Together these experiments represent a clear in vitro counterpart to the effects observed in the muscle injury model, showing the key role of ANXA1 acting through FPR2/ALX to drive a shift in macrophage phenotype toward a pro-resolving and reparative polarization.

### Exogenous ANXA1 treatment stimulates AMPK activation through FPR2/ALX.

The enzyme 5′-adenosine monophosphate–activated protein kinase (AMPK) plays a critical role in macrophage phenotype skewing and is necessary for efficient regeneration after skeletal muscle damage (13). We queried whether this signaling pathway would underlie the effects of hrANXA1 on human macrophage phenotype and indeed muscle repair in vivo.

**Figure 4:**
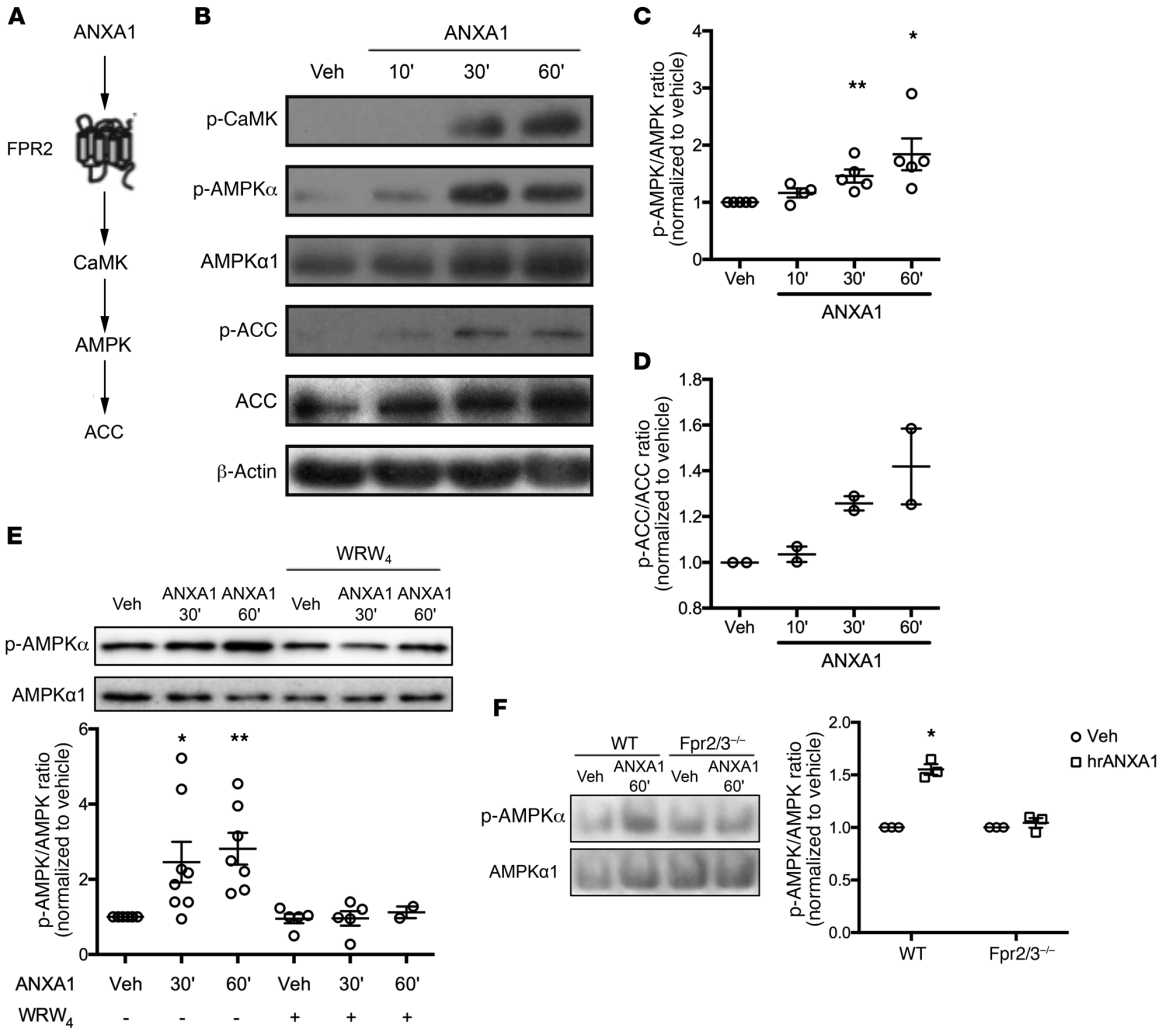
The ANXA1–FPR2/ALX pathway activates the AMPK signaling cascade in human and murine macrophages. (**A**) Schematic representation of the ANXA1–FPR2/ALX signaling cascade. (**B**–**D**) Western blot analysis of phosphorylated CaMK, AMPKα1, and acetyl-CoA carboxylase (ACC) in human PBMC–derived macrophages treated with hrANXA1. Shown are representative blots (**B**) and quantification of p-AMPKα1/AMPKα1 (**C**) and p-ACC/ACC (**D**) ratios. (**E**) Representative Western blot (top) and quantification (bottom) of p-AMPKα/AMPKα1 ratio in human PBMC–derived macrophages treated by hrANXA1 in the presence or absence of 10 μM WRW_4_. (**F**) Representative Western blot (left) and quantification (right) of p-AMPKα/AMPKα1 ratio in WT or Fpr2/3^–/–^ murine bone marrow–derived macrophages treated with hrANXA1. Results are mean ± SEM of at least 2 (**D**) or 3 (**C**, **E**, and **F**) independent experiments. *P < 0.05, **P < 0.01 vs. vehicle.

Exposure of human PBMC–derived macrophages to hrANXA1 activated a number of components of the AMPK signaling pathway, promoting phosphorylation of Ca^2+^/calmodulin-dependent protein kinase II, AMPKα itself, and its downstream effector acetyl-CoA carboxylase (Figure 4, A and B). Interestingly, phosphorylation of these proteins in macrophages only occurred to an appreciable degree following exposure to hrANXA1 for 30–60 minutes (Figure 4, B–D), in contrast to early MAP kinase signaling previously reported in human monocytes (18). That activation of AMPKα depended on binding of hrANXA1 to FPR2/ALX was confirmed through analysis of the effects of the antagonist WRW_4_, which abrogated hrANXA1-evoked AMPKα phosphorylation in human macrophages (Figure 4E). The specificity of hrANXA1 action through its human receptor FPR2/ALX was also determined with Fpr2/3^–/–^ cells lacking the ortholog of the human FPR2/ALX (27). Analyses of murine bone marrow–derived macrophages (BMDMs) from WT and Fpr2/3^–/–^ animals confirmed the pivotal role of this receptor: while treatment with hrANXA1 induced phosphorylation of AMPKα in WT cells, this response was absent in macrophages from Fpr2/3^–/–^ mice (Figure 4F).

### AMPK activation is required for macrophage phenotype conversion induced by ANXA1.

**Figure 5:**
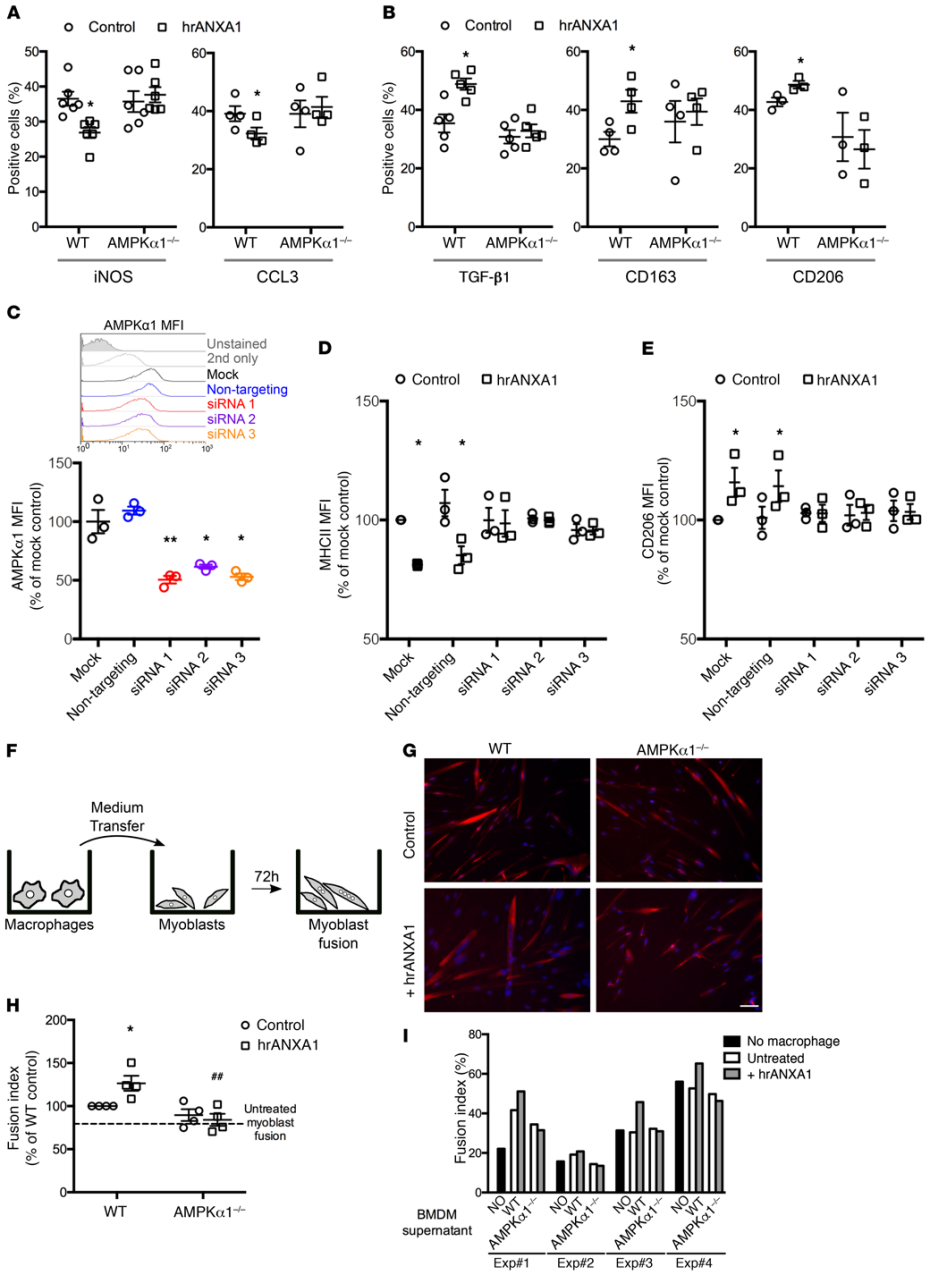
Null or reduced AMPK expression affects ANXA1-mediated macrophage polarization. (**A** and **B**) Primary macrophages derived from WT or AMPKα1^–/–^ mice were treated with 10 nM hrANXA1, and the percentage of cells expressing the proinflammatory markers iNOS and CCL3 (**A**) and the antiinflammatory markers TGF-β1, CD163, and CD206 (**B**) was determined by immunofluorescence. (**C**–**E**) Human PBMC–derived macrophages were transfected by a nontargeting or 3 different AMPKα1-targeting siRNAs and treated with 10 nM hrANXA1 for 24 hours. AMPKα1 protein level was determined by FACS (**C**), and the MFI units of the proinflammatory MHCII (**D**) and antiinflammatory CD206 (**E**) markers were measured by flow cytometry. (**F**–**I**) Conditioned medium produced by murine macrophages was transferred onto murine primary myoblasts, and their fusion was measured by immunofluorescence. (**F**) Experimental setup. (**G**) Representative images of desmin (red) and Hoechst (blue) labeling of myoblast cultures. Scale bar: 50 μm. (**H** and **I**) Fusion index calculated after desmin labeling. Shown are the means ± SEM of the independent experiments relative to the WT control (**H**) and the raw results of each individual replicate (**I**). Results are mean ± SEM of at least 3 independent experiments. *P < 0.05, **P < 0.01 vs. mock or control; ^##^*P* < 0.01 vs. WT.

We investigated the relationship between FPR2/ALX–mediated AMPK activation and the shift in macrophage phenotype induced by hrANXA1 treatment through analyses in primary BMDMs taken from WT mice and animals lacking the key catalytic α1 subunit of AMPK (28). While treatment of WT macrophages with hrANXA1 (10 nM, 6 hours) reduced the percentage of cells positive for the proinflammatory markers iNOS and CCL3, this response was notably absent in cells from AMPKα1^–/–^ mice (Figure 5A). Correspondingly, hrANXA1 treatment augmented the proportion of WT cells expressing the antiinflammatory markers TGF-β1 (notably at variance from human macrophages), CD163, and CD206, but this did not occur in cells lacking AMPKα1 (Figure 5B). Together, these data indicate activation of AMPKα1 as a key step in the macrophage phenotype shift induced by ANXA1.

To confirm these findings in human cells, we used an RNA interference approach, transfecting primary PBMC-derived macrophages with 3 distinct siRNA constructs targeting the AMPKα1 subunit (Figure 5C). Treatment of M1-like proinflammatory macrophages for 6 hours with 10 nM ANXA1 reduced the expression of MHCII and augmented that of CD206 in mock-transfected cells and in cells bearing a nontargeting siRNA construct; this effect was absent in cells transfected with any of 3 different siRNA constructs targeting human AMPKα1 (Figure 5, D and E, and Supplemental Figure 7A).

### Adult myogenesis is dependent on ANXA1/AMPK signaling in vitro.

Together, data from the experiments with human and mouse macrophages make a compelling case that macrophage phenotype shifting can be induced through an ANXA1/FPR2/AMPK cascade. To investigate whether this process underlies differences in recovery from cardiotoxin-induced muscle lesions seen between WT and AnxA1^–/–^ and Fpr2/3^–/–^ mice, we made use of an established model of in vitro muscle repair (13, 29).

We used an in vitro model of adult myogenesis in which conditioned medium from primary BMDMs was used to stimulate primary murine myoblasts for 72 hours, quantifying the proportion of multinucleated myotubes (Figure 5F). As such, this in vitro setting recapitulates the processes of adult myogenesis (activation, differentiation, migration, and fusion of muscle cells) that occur during skeletal muscle regeneration (13, 29). Conditioned medium from ANXA1-treated WT macrophages augmented the myotube fusion index (Figure 5, G–I). This response was not observed when myotubes were treated with conditioned medium from ANXA1-treated AMPKα1^–/–^ macrophages (Figure 5, G–I). Direct addition of ANXA1 (10 nM) to myoblast cultures did not regulate the extent of cell fusion (Supplemental Figure 7B).

## Discussion

The timely resolution of inflammation is a fundamental requirement for restoring homeostasis following infection or damage, and its failure contributes significantly to numerous chronic inflammatory pathologies. Macrophages are key players in this process, given their ability to transition from generally proinflammatory to antiinflammatory phenotypes (7). Substantial effort has gone into deciphering the complex signals underpinning macrophage plasticity; numerous soluble mediators have been implicated (11), but the mechanistic link between these factors and changes in phenotype remains poorly understood, especially when investigated in settings restricted to specific tissues. In the current study, we have used a well-characterized model of muscle injury and recovery (13) to identify the ability of myeloid cell–derived ANXA1 to promote an antiinflammatory macrophage phenotype, promoting resolution and tissue repair. Moreover, we show the actions of this protein to be mediated through the cell surface receptor FPR2/ALX and downstream activation of the intracellular signaling molecule AMPK, mechanistically linking external and intracellular pro-resolving signals governing macrophage phenotype.

These data reinforce the identification of the ANXA1–FPR2/ALX pair as a major endogenous driver of inflammatory resolution, and add to its known roles in regulating neutrophil apoptosis (17), efferocytosis (20, 30), and the recruitment of monocytes to inflammatory sites (18). Notably, despite muscle tissue itself beginning to express ANXA1 during tissue repair, as noted previously (31), experiments with chimeric mice confirmed that in our experimental settings the most important source of the protein to enable muscle repair remains the myeloid cells themselves. Moreover, although previous studies have highlighted a direct plasma membrane action of extracellular ANXA1 in myoblast fusion in vitro (32, 33), our experiments indicate that Fpr2/3, the receptor target of ANXA1, is not expressed by stromal and parenchymal cells in vivo. This suggests that, at least in the context of skeletal muscle regeneration after cardiotoxin application, the action of ANXA1 is mainly mediated by myeloid cells, which in turn regulate myogenic cells. While our studies do not absolutely identify whether neutrophils or macrophages are the primary source of endogenous ANXA1 within the injured tissue, neutrophils contain substantial amounts of this mediator (34), which we have previously shown to be a major monocyte chemoattractant (18), suggesting that the protein — produced at an early stage by and released from infiltrating neutrophils (35) — may act to both recruit monocytes and promote a pro-resolving phenotype in the resulting macrophages. Irrespective of the cell that is the source of ANXA1, the dependence on the presence of ANXA1-expressing leukocytes for efficient macrophage phenotypic conversion is another example of how “the beginning programs the end” in resolution (36), emphasizing the finely tuned nature of the acute inflammatory response and its ability to encode its own termination.

Besides highlighting the role of ANXA1 as a regulator of inflammatory resolution (37), these data add further weight to the importance of its receptor FPR2/ALX in this process, identifying it as a major determinant for induction of a pro-resolving macrophage phenotype through AMPK activation. Notably, macrophage FPR2/ALX expression declined during the course of the response to muscular injury; congruently, FPR2/ALX expression was significantly lower in M2-like macrophages compared with M1-like–phenotype cells in vitro. This modulation of receptor expression may reflect a mechanism whereby once the macrophage is polarized toward a reparative phenotype, the utility of the FPR2/ALX signaling is no longer necessary.

There is considerable redundancy in the mediators known to induce the conversion of macrophage phenotypes, a feature that is perhaps expected given the importance of this cellular process to promote restoration of homeostasis after infection or damage; these factors include immune complexes, apoptotic cells, and specific cytokines (38). Notably, however, a significant proportion of these macrophage-polarizing mediators derive from the adaptive arm of the immune response, particularly from Th2 lymphocytes (11). Herein, we identify signaling components derived from the innate side of the immune system to promote resolution. Moreover, these results support the concept that it is crucial to decipher mediators and signal(s) operative in specific tissues and organs to control macrophage polarization, hence to regulate the whole process of resolution and repair.

Our data associate activation of FPR2/ALX and the major regulator of cellular metabolism AMPK, showing the central involvement of the FPR2/ALX–stimulated AMPK pathway in the induction of a pro-resolving macrophage phenotype. The precise mechanism linking AMPK activation with a change in phenotype is as yet unclear, but there is increasing evidence that changes in the metabolic status of immune cells can affect their inflammatory activity (39, 40). Proinflammatory dendritic cells and T lymphocytes are characterized by high levels of glycolysis, akin to the Warburg metabolic shift described in cancer (41), whereas immune cells with an antiinflammatory or pro-resolving profile tend to exhibit greater mitochondrial respiration (13, 42, 43). The mechanistic details of how such changes in metabolic phenotype relate to immune function, and indeed whether these differences reflect or drive immunophenotype, are not fully elucidated (44), but it is notable that AMPK is a significant promoter of mitochondrial respiration and a regulator of glycolysis through modulation of lactate dehydrogenase activity (45), driven by its ability to respond to energy debt and an increased AMP/ATP ratio (46). Activation of this pathway is ideally placed to induce the metabolic phenotype most closely associated with antiinflammatory macrophage activation, but this hypothesis requires further investigation.

AMPK is involved in different cellular mechanisms that regulate skeletal muscle homeostasis (47). Our previous findings highlighted the importance of crosstalk between AMPK and the mTOR signaling pathway for the control of muscle cell size in the adaptive response of skeletal muscle (48–51). Moreover, we have recently shown that AMPKα1, activated following phagocytosis, is crucial for macrophage skewing from a pro- to an antiinflammatory phenotype during resolution (13), demonstrating that the CAMKKII/AMPKα1 cascade within macrophages is required for proper and complete skeletal muscle regeneration. Antiinflammatory macrophages promote myogenic differentiation and fusion (29, 52, 53), a finding of importance for skeletal muscle regeneration, in which a sequential presence of proinflammatory, then antiinflammatory, cells is necessary for an efficient regeneration process (23, 24). We could demonstrate that macrophages conditioned by ANXA1, but not the soluble mediator itself, promoted myogenic fusion.

In summary, we present a mechanism governing the conversion of proinflammatory macrophages to a pro-resolving phenotype, linking leukocyte-derived ANXA1, FPR2/ALX, and intracellular AMPK activation. Altogether, these data identify the ANXA1–FPR2/ALX pathway as a pivotal regulator of inflammatory resolution with nonredundant downstream actions on tissue repair. As such, ANXA1 and FPR2/ALX represent suitable targets for therapeutic exploitation for the innovative treatment of pathologies characterized by chronic, nonresolving inflammation.

## Methods

### Animals.

Male C57BL/6 mice, male *alx/fpr2/3^GFP/GFP^* mice (referred to as Fpr2/3^–/–^) bearing a knocked-in green fluorescent protein (27), and male *AnxA1^–/–^* mice (54), aged 10 weeks, were used for in vivo experiments. Both transgenic strains were fully backcrossed onto a C57BL/6 genetic background.

### Production of recombinant human ANXA1.

Human recombinant annexin A1 (hrANXA1) was produced by a prokaryotic expression system and purified essentially as described previously (55). Briefly, cDNA for human ANXA1 was inserted into the expression vector pQE30Xa (Qiagen Ltd.) and transfected into *E. coli* (SG13009 pREP4, Novagen), which were then grown in Luria-Bertani broth medium supplemented with ampicillin (50 μg/mL; Roche), kanamycin (30 μg/mL; Thermo Fisher Scientific), and 0.5% glycerol. Protein overexpression was initiated by addition of 5 mM isopropyl β-d-1-thiogalactopyranoside (Eurogentec), and proteins were purified by immobilized metal ion affinity chromatography. Purity and homogeneity were assessed by SDS-PAGE, Western blotting, and MALDI-TOF/TOF analysis. Endotoxin was determined with the Endosafe-PTS (FDA-licensed *Limulus* amebocyte lysate cartridge from Charles River Laboratories) according to the manufacturer’s protocol. HrANXA1 contained less than 0.2 U endotoxin per mg hrANXA1 protein.

### Skeletal muscle injury.

Skeletal muscle injury was caused by intramuscular injection of cardiotoxin (Latoxan) in the tibialis anterior (TA) muscle of male animals, as described previously (13). TA muscles were injected with cardiotoxin (50 μL per TA, 12 μM); 1, 2, 7, 14, or 28 days after lesioning, animals were killed by exposure to CO_2_ or cervical dislocation after isofluorane anesthesia. TAs were isolated and snap-frozen in liquid nitrogen–cooled isopentane for storage and later analysis. Only muscles harboring more than 90% of myofibers with centrally located nuclei were considered for analysis.

### Murine BMDMs.

Bone marrow–derived macrophages (BMDMs) were prepared from adult male WT Sv129/C57BL/6 and *Prkaa1^–/–^* mice (referred to as AMPKα1^–/–^; ref. 28). Mice were killed by cervical dislocation under isoflurane anesthesia, and marrow was flushed from tibiae and femurs. Cells were plated, washed, and grown for 6–7 days in DMEM High Glucose High Pyruvate, 20% heat-inactivated FCS (Thermo Fisher Scientific), 30% L929 cell conditioned medium, 1% amphotericin B (2.5 μg/mL; Thermo Fisher Scientific), and 100 μg/mL streptomycin (Thermo Fisher Scientific). For phenotypic characterization, BMDMs were treated for 6 hours in the presence or absence of 10 nM hrANXA1 and fixed for 10 minutes in 4% formaldehyde, permeabilized for 10 minutes in PBS with 0.5 % Triton X-100, and blocked for 1 hour in PBS with 4% BSA. They were then labeled overnight at 4°C with anti–NOS-2 (ab15323, Abcam), anti-CCL3 (ab32609, Abcam), anti–TGF-β1 (ab64715, Abcam), anti-CD163 (sc-20066, Santa Cruz Biotechnology), and anti-CD206 (sc-58987, Santa Cruz Biotechnology), followed by incubation for 1 hour at 37°C with Cy3-conjugated secondary antibodies (Jackson ImmunoResearch Laboratories Inc.). Cells were stained with Hoechst (MilliporeSigma) and mounted in Fluoromount (Interchim), and pictures were taken on an Axio Imager.Z1 (Zeiss) at ×20 magnification connected to a CoolSNAP MYO CCD Camera (Photometrics).

### In vitro model of adult myogenesis.

Macrophages were obtained from bone marrow precursor cells extracted from 4 distinct mice that were cultured in DMEM containing 20% FBS and 30% conditioned medium of L929 cell line (enriched in CSF-1) for 7 days. Macrophages were activated with hrANXA1 for 3 days (10 nM) in DMEM containing 10% FBS. After the washing steps, serum-free DMEM was added for 24 hours to obtain macrophage-conditioned medium. Murine myogenic precursor cells (MPCs) were obtained from TA muscle isolated from 4 mice and cultured using standard conditions in DMEM/F12 medium (Gibco Life Technologies) containing 20% heat-inactivated FBS and 2% G/Ultroser (Pall Inc.). MPCs were seeded at 30,000 cells/cm^2^ on Matrigel (diluted 1:10) and incubated for 3 days with conditioned medium containing 2% heat-inactivated horse serum. In the case of direct treatment of MPCs, cells were directly incubated with 10 nM hrANXA1 for 3 days in the presence of 2% heat-inactivated horse serum. Cells were then incubated with an anti-desmin antibody (ab32362, Abcam), followed by a Cy3-conjugated secondary antibody (Jackson ImmunoResearch Laboratories Inc.) (13, 29). Cells were stained with Hoechst (Sigma-Aldrich) and mounted in Fluoromount (Interchim), and pictures were taken on Axio Observer.Z1 (Zeiss) at ×20 magnification connected to a CoolSNAP HQ2 CCD Camera (Photometrics).

### Bone marrow transplantation.

Bone marrow transplantation was performed as previously described (13, 56). Total bone marrow cells were isolated by flushing of the tibiae and femurs of 8- to 20-week-old donor mice (WT or AnxA1^–/–^ males) with RPMI 1640/10% FBS. They were transplanted into 8- to 12-week-old recipient CX3CR1-GFP^+/–^ males (monocytes/macrophages expressing GFP) previously lethally irradiated for 10 minutes with a dose of 0.85 Gy/min in an X-RAD 320 (Precision X-Ray). Total bone marrow cells were injected (10^7^ cells diluted in 100 μL of RPMI 1640/50% mouse serum) into the retro-orbital vein of recipient mice. After transplantation, mice were fed with ciprofloxacin (10 mg/kg/d) in the drinking water for 3 weeks. Engraftment efficiency was determined by FACS analysis on peripheral blood 5 weeks after the transplantation and on bone marrow when mice were sacrificed. Briefly, red cells were lysed with ACK buffer, and leukocytes were incubated with FcR Blocking Reagent (Miltenyi Biotec) for 20 minutes at 4°C. Finally, cells were labeled with an APC-conjugated anti-CD115 antibody for 30 minutes at 4°C and analyzed on a BD FACSCanto II (BD Biosciences). DAPI was used as viability marker. Engraftment was determined as the percentage of monocytes not expressing GFP.

### Human peripheral blood–derived macrophages.

Human cells were prepared according to an approved protocol (East London and the City Research Ethics Committee and Queen Mary University of London [QMUL] Animal Welfare & Ethical Review Board; 06/Q605/40; P/00/029). Peripheral blood was collected from 6 different healthy volunteers by intravenous withdrawal in 3.2% sodium citrate solution (1:10). PBMCs were isolated by density centrifugation on a Histopaque-1077 gradient (Sigma-Aldrich) according to the manufacturer’s instructions, and were plated in RPMI 1640 for 1 hour. Cells were washed 3 times with ice-cold PBS without Ca^2+^/Mg^2+^ to remove lymphocytes, and adherent cells were incubated in RPMI 1640 containing 20% heat-inactivated FCS for 14 days.

### Histological and immunohistochemical analysis.

For histological analysis, muscles were harvested, snap-frozen in liquid nitrogen–chilled isopentane, and kept at –80°C until use. Cryosections (10 μm) were prepared for H&E or Sudan Black staining. Stained sections were scanned using an Axio Scan.Z1 (Zeiss) with a ×20 objective and a 3 CCD HV-F 2025 CL camera (Hitachi), and Sudan Black–labeled areas were quantified with ImageJ software (NIH). Briefly, images were converted into 8-bit binary image using a Yen threshold filter, and black pixels were enumerated and expressed as a percentage of the total pixels in the muscle section. Fluorescence immunohistochemical analysis was performed according to standard procedures. Briefly, transverse muscle cryosections (10 μm) were postfixed by incubation for 15 minutes in 4% formaldehyde, blocked, and immunostained using primary antibodies directed against Ly6G (1:100; 127602, BioLegend), F4/80 (1:200; 123102, BioLegend), ANXA1 (1:1000; 71-3400, Thermo Fisher Scientific), or Fpr2/3 (1:100; sc-18191-R, Santa Cruz Biotechnology). Secondary antibodies were Alexa Fluor 488–conjugated (AF488-conjugated) or AF594-conjugated goat anti-rabbit or anti-rat IgG (1:300; Invitrogen, Thermo Fisher Scientific). Sections were counterstained with DAPI, mounted, and examined using a TCS SP5 confocal laser scanning microscope (Leica Microsystems) fitted with 405-nm, 488-nm, and 594-nm lasers and attached to a Leica DMI6000CS inverted microscope fitted with a ×40 objective lens (NA 0.75 mm; working distance, 0.66 mm). Images were captured with Leica LAS AF 2.6.1 software and analyzed using ImageJ 1.51w software (NIH).

### In vivo macrophage phenotype analysis.

Macrophage phenotype was analyzed as previously described (13). Briefly, CD45^+^ cells were isolated from regenerating TA muscle using magnetic beads conjugated to anti-CD45 antibody (Miltenyi Biotec) and then incubated with Fc block (Miltenyi Biotec) for 30 minutes at 4°C. Finally, CD45^+^ cells were stained with antibody against Ly6C/G (17-5931-82, eBioscience) and against F4/80 (12-4801-82, eBioscience). Percentages of Ly6C/G^hi^F4/80^lo^ and Ly6C/G^neg^F4/80^hi^ cells were calculated among total F4/80^+^ cells after analysis by flow cytometry with a FACSCalibur instrument (BD Biosciences) and FlowJo version 9.2 analysis software as described below.

### AMPKα1 siRNA.

Primary human PBMC–derived macrophages were transfected with 1 of 3 different commercial siRNA sequences designed to target AMPKα1 or an Allstars negative control siRNA sequence using Hiperfect transfection reagent according to the manufacturer’s instructions (final concentration 2 nM; all Qiagen GmbH), alongside mock-transfected cells. After 48 hours, cells were analyzed for phenotypic conversion following hrANXA1 treatment (6 hours, 10 nM). A proportion of cells were analyzed for AMPKα1 expression by Western blot as described below or by flow cytometry. Briefly, 48 hours after siRNA transfection, cells were detached, fixed by incubation in 4% formaldehyde in PBS at 4°C for 10 minutes, and permeabilized by resuspension with vortexing in ice-cold methanol. Surface Fcγ receptors were blocked by incubation for 20 minutes at 4°C with IgG block (Thermo Fisher Scientific), then incubated for 30 minutes at 4°C with a rabbit polyclonal antibody raised against the human AMPKα1 subunit (1:1000; 2795, Cell Signaling Technology), followed by staining with an AF488-conjugated goat anti-rabbit polyclonal secondary antibody (1:500; A-11008, Thermo Fisher Scientific). Cells were analyzed on a FACSCanto II flow cytometer (BD Biosciences) using FlowJo 8.8.2 analysis software (Tree Star Inc.). A total of 10,000 singlet events were analyzed for each sample.

### Human macrophage flow cytometry analysis.

Primary human PBMC–derived macrophages were labeled with FITC-conjugated mouse monoclonal anti-MHCII (11-9956-42, Thermo Fisher Scientific) and APC-conjugated mouse monoclonal anti-CD206 (17-2069-42, Thermo Fisher Scientific) or isotype controls (all Thermo Fisher Scientific) according to the manufacturer’s protocols. In all cases, 20,000 events were acquired using a FACSCalibur flow cytometer (BD Biosciences), and analyzed using FlowJo 9.2 analysis software (Tree Star Inc.). In some cases, macrophages were analyzed for surface expression of FPR2/ALX; surface Fcγ receptors were blocked by incubation for 20 minutes at 4°C with IgG block (Thermo Fisher Scientific), followed by incubation for 30 minutes at 4°C with mouse monoclonal anti–FPR2/ALX (1 μg/106 cells; GM1D6, Aldevron), then incubation for 30 minutes at 4°C with secondary antibody (AF488-conjugated goat anti-mouse, 1:300; Thermo Fisher Scientific).

### Western blot analysis.

Mouse muscle tissue and human macrophage samples were homogenized in RIPA buffer containing phosphatase and protease inhibitor cocktail (Thermo Fisher Scientific). Samples boiled in 6× Laemmli buffer were subjected to standard SDS-PAGE (10%) and electrophoretically blotted onto Immobilon-P polyvinylidene difluoride membranes (Millipore). Membranes were incubated with antibodies raised against human phospho–Ca^2+^/calmodulin-dependent kinase (12716, Cell Signaling Technology), phospho-AMPKα (2531, Cell Signaling Technology), AMPKα1 (2795, Cell Signaling Technology), phospho–acetyl-CoA carboxylase (3661, Cell Signaling Technology), acetyl-CoA carboxylase (3662, Cell Signaling Technology) (all 1:1000), ANXA1 (1:1000; 71-3400, Thermo Fisher Scientific, UK), or β-actin (1:10,000; A5316, Sigma-Aldrich) in Tris-buffered saline solution containing 0.1% Tween-20 and 5% (wt/vol) nonfat dry milk overnight at 4°C. Membranes were washed for 30 minutes with Tris-buffered saline solution containing 0.1% Tween-20, with the solution being changed at 10-minute intervals; membranes were then incubated with secondary antibody (HRP-conjugated goat anti-mouse, 1:5000; Thermo Fisher Scientific) for 2 hours at room temperature. Proteins were then detected using an enhanced chemiluminescence detection kit and visualized on Hyperfilm (Amersham Biosciences). Films were digitized and analyzed using ImageJ 1.51w software (NIH).

### Quantitative reverse transcriptase PCR.

Total RNA was prepared from primary human PBMC–derived macrophages using TRIzol reagent (Life Technologies Ltd.) and then reverse-transcribed with SuperScript III reverse transcriptase (Life Technologies Ltd.) according to the manufacturer’s protocols. Resultant cDNA was then analyzed by real-time PCR in duplicate, using the Quantitect primer system (primer sets: FPR2/ALX QT00204295, IL-10 QT00041685, NOS-2 QT00068740, TNF-α QT00029162, and TGF-β1 QT00000728; all Qiagen Ltd.) and Power SYBR Green PCR Master Mix (Applied Biosystems, Thermo Fisher Scientific). Reactions were performed in 384-well format using the ABI PRISM 7900HT Sequence Detection System. The PCR conditions consisted of 95°C for 15 minutes, then 40 cycles of 95°C for 15 seconds, 55°C for 30 seconds, and 72°C for 30 seconds, with a dissociation step of 95°C for 15 seconds, 60°C for 15 seconds, and 95°C for 15 seconds included after the PCR reaction to confirm the absence of nonspecific products. Data were acquired and analyzed with SDS 2.3 (Applied Biosystems, Thermo Fisher Scientific); fold change was calculated as 2^−ΔΔCt^.

For AnxA1 and Fpr2/3 quantitative reverse transcriptase PCR analysis, cell populations were FACS-sorted from mouse TA muscles as previously described (57) using a FACSAria II cell sorter (BD Biosciences). Total RNA was isolated using NucleoSpin RNA Plus XS kit (Macherey-Nagel) and retro-transcribed into cDNA using SuperScript II Reverse Transcriptase. Quantitative PCR was performed in triplicate on a CFX Connect Real-Time PCR Detection System (Bio-Rad) using LightCycler 480 SYBR Green I Master (Roche Diagnostics). Calculation of relative expression was determined by Bio-Rad CFX Manager software, and fold change was normalized as normalized relative quantity (NRQ) (or ΔΔCq) for each series:

(Equation 1)
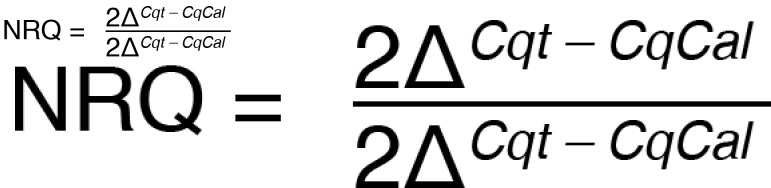


where *T* is the target sample, *Cal* is the calibrator value (i.e., the mean of all sample Cqs of the series), and *R* is the housekeeping gene cyclophilin A. Sequences of primers used were as follows: 5′-GCACTCCAGCTTTCTTTGCC-3′ (AnxA1 forward), 5′-AATTTCCGAACGGGAGACCA-3′ (AnxA1 reverse), 5′-ACACCACAGGAACCGAAGAG-3′ (Fpr2/3 forward), 5′-TGGAGACAACCACCATTGAGA-3′ (Fpr2/3 reverse), 5′-GTGACTTTACACGCCATAATG-3′ (cyclophilin A forward), and 5′-ACAAGATGCCAGGACCTGTAT-3′ (cyclophilin A reverse).

### Statistics.

All quantified in vitro data are derived from at least 3 independent donors, with experiments performed in triplicate, and are expressed as mean ± SEM. Murine in vivo experiments were performed with a group size of *n* = 6, sufficient to identify a 20% effect size with a power of 0.8, and are expressed as mean ± SEM. All mice were randomly allocated to groups, and analysis was performed blinded to experimental condition. Data were analyzed by 1- or 2-way ANOVA as appropriate, with post hoc comparison using Tukey’s honestly significant difference test. For murine in vitro experiments, at least 3 independent experiments were performed, and statistical significance was determined using Student’s *t* test. In all cases, *P* less than 0.05 was taken as indicating statistical significance.

### Study approval.

All procedures were performed under the UK Animals (Scientific Procedures) Act, 1986, in the United Kingdom or in compliance with European legislation in France. Animal facilities were fully licensed by relevant national authorities, and protocols were validated by local ethical committees (CEEA55, Université Claude Bernard Lyon 1, and Animal Welfare and Ethical Review Body, in QMUL).

## Author contributions

SM, TG, GJ, JG, MT, and RM performed TA lesioning and analysis. GJ and RM produced and analyzed chimeric mice. SM, TG, and JETK performed analysis of human macrophages. GJ, JG, MT, and RM performed analysis of AMPKα1-null mice. TD and RM performed murine macrophage and myoblast fusion analysis. CPR produced and provided human recombinant ANXA1. SM, GJ, BC, MP, and RM conceived and designed the study; all authors contributed to the drafting of the paper. TG made the initial core findings, whereas SM and GJ played a larger role in later mechanistic studies and in manuscript completion and were listed according to their affiliations to balance with the order of the senior coauthorship (MP and RM).

## Supplementary Material

Supplemental data

## Version 1 02/04/2020

Electronic publication

## Version 2 03/02/2020

Print issue publication
