# Supplementary material for: Annexin A1 drives macrophage skewing to accelerate muscle regeneration through AMPK activation
Source: J Clin Invest. 2020 Feb 4;130(3):1156–67. doi: 10.1172/JCI124635 (PMC7269594; doi:10.1172/JCI124635)
Supplement: Supplemental data [file jci-130-124635-s150.pdf]

Suppl Fig 1. Related to Figure 1.

(A) HE staining of non-injured Wild-Type and AnxA1$^{-/-}$ *Tibialis Anterior* (TA) muscles. White bar = 50 µm. (B) Myofibre cross-sectional area of non-injured Wild-Type and AnxA1$^{-/-}$ TA muscles. Shown is

the mean +/- SEM of 8 muscles. (C) Number of nuclei *per* myofibre in muscles 7 days post-CTX injury. (D) Representative images of Sudan black staining of TA muscles from Wild-Type, $AnxA1^{-/-}$ and $Fpr2/3^{-/-}$ mice 28 days post-cardiotoxin injury. (E) Representative FACS plots of F4/80 and Ly6C/G markers in TA of Wild-Type, $AnxA1^{-/-}$ and $Fpr2/3^{-/-}$ mice 2 days post-cardiotoxin injury. (F-G) Macrophage subtypes analysis in Wild-Type and $Fpr1^{-/-}$ muscles 2 days post-CTX injury. Shown are the percentage of pro- and anti-inflammatory macrophages within the $F4/80^{+}$ population (F) and the resolution index (G). Results are mean ± SEM of at least three animals. *$p<0.05$ *versus* Wild-Type.

Suppl Fig 2. Related to Figure 2. Gating strategy to isolate cell populations from *Tibialis anterior* muscle.

TA muscles were digested with Collagenase B/Dispase and cells were separated by magnetic beads based on their CD45 expression level. (A) $CD45^{pos}$ cells were blocked with anti-FcgRII/III and labelled with CD45, CD64 and Ly6C antibodies to isolate Neutrophils (Neut) and $Ly6C^{pos}$ and $Ly6C^{neg}$ macrophages (Mac). Shown are representative plots for day 4 post-CTX injury together with purity check for recovered populations (coloured boxes). Numbers represent sorting purity calculated as percentage of live cells. (B) $CD45^{neg}$ cells were labelled with CD45, CD31, Sca-1, α7-integrin and CD34 antibodies to isolate endothelial cells (EC), FAPs (FAP) and satellite cells (SAT). Shown are representative plots at day 4 (A) and day 1 (B) post-CTX injury, together with purity check for recovered populations (coloured boxes). Numbers represent sorting purity calculated as percentage of live cells.

Suppl Fig 3. Related to Figure 2. Immune cell recruitment and pattern of AnxA1 expression in cardiotoxin-induced muscle injury and repair.

(A) Anxa1 mRNA level measured by RT-qPCR on sorted cell populations from *Tibialis Anterior* muscle after cardiotoxin injury. (B-D) Immunofluorescence analysis of ANXA1 protein in *Tibialis Anterior* muscle after cardiotoxin injury. Images show co-localisation of ANXA1 protein with $Ly6G^{+}$ (B) and $F4/80^{+}$ (C) cells. White bar = 50 µm. (D) Quantification of $F4/80^{+}$ $ANXA1^{+}$ cells in *Tibialis Anterior* (TA) muscle after cardiotoxin (CTX) injury.

Suppl Fig 4. Related to Figure 2. Immune cell recruitment and Fpr2/3 expression in cardiotoxin-induced muscle injury and repair.

(A) Fpr2/3 mRNA level measured by RT-qPCR on sorted cell populations from *Tibialis Anterior* muscle after cardiotoxin injury. (B-C) Quantification of $F4/80^{+}$ $ANXA1^{+}$ cells in *Tibialis Anterior* (TA) muscle after cardiotoxin (CTX) injury. Representative immunofluorescence analysis of Fpr2/3 protein in TA

muscle after cardiotoxin (CTX) injury (B) and quantification of the percentage of F4/80$^+$ cells expressing Fpr2/3 (C). White bar = 50 µm. Results are means ± SEM of three animals. *p<0.05 and ***p<0.001 *versus* Day 0.

Suppl Fig 5. Related to Figure 2. BMT controls

(A) Body weight follow-up, represented as percentage of Day 0, of mice after irradiation and bone marrow transplantation. Results are means ± SEM of at least six animals. *p<0.05 *versus* Day 0. (B-C) Representative FACS plots (B) and quantification (C) of the GFP$^+$ monocytes in the bone marrow of the sacrificed animals. (D-E) Representative FACS plots (D) and quantification (E) of the percentage of macrophages with host or donor origin based on their GFP expression. Shown are TA muscles between day 2 and day 8 post-CTX injury.

Suppl Fig 6. Related to Figure 3. FPR2/ALX expression varies following human macrophage polarisation.

Human primary macrophages were polarised into M1 or M2 macrophages with IFNg and IL-4, respectively (A-C) Flow cytometry analysis of FPR2/ALX expression. Shown are representative FACS plots (A), percentage of cells expressing FPR2/ALX (B) and FPR2/ALX MFI (C). (D) RT-qPCR analysis of FPR2/ALX mRNA level. Results are means +/- SEM of at least four independent experiments. *p < 0.05 *versus* M0 (non-activated).

Suppl Fig 7. Related to Figure 5.

(A) Human PBMC-derived macrophages were transfected by a non-targeting or three different AMPKα1-targeting siRNAs and treated with 10 nM hrANXA1 for 24 h. AMPKα1 protein level was determined by western blot. (B) Primary myoblasts were differentiated for 3 days in presence of 10 nM hrANXA1 or conditioned medium produced by murine macrophages treated by 10 nM hrANXA1. Each dot corresponds to an independent experiment.

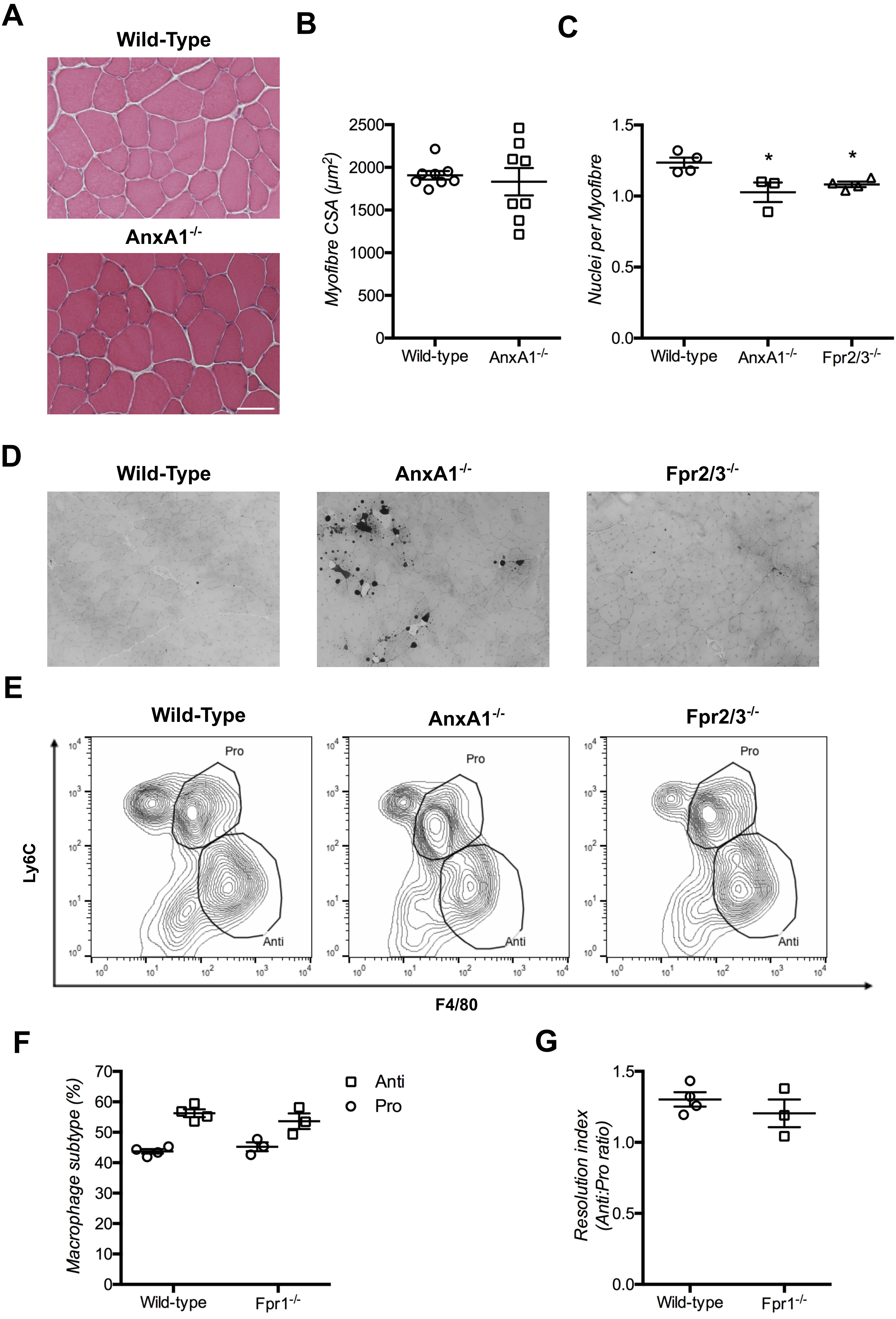

Supplementary Figure 1

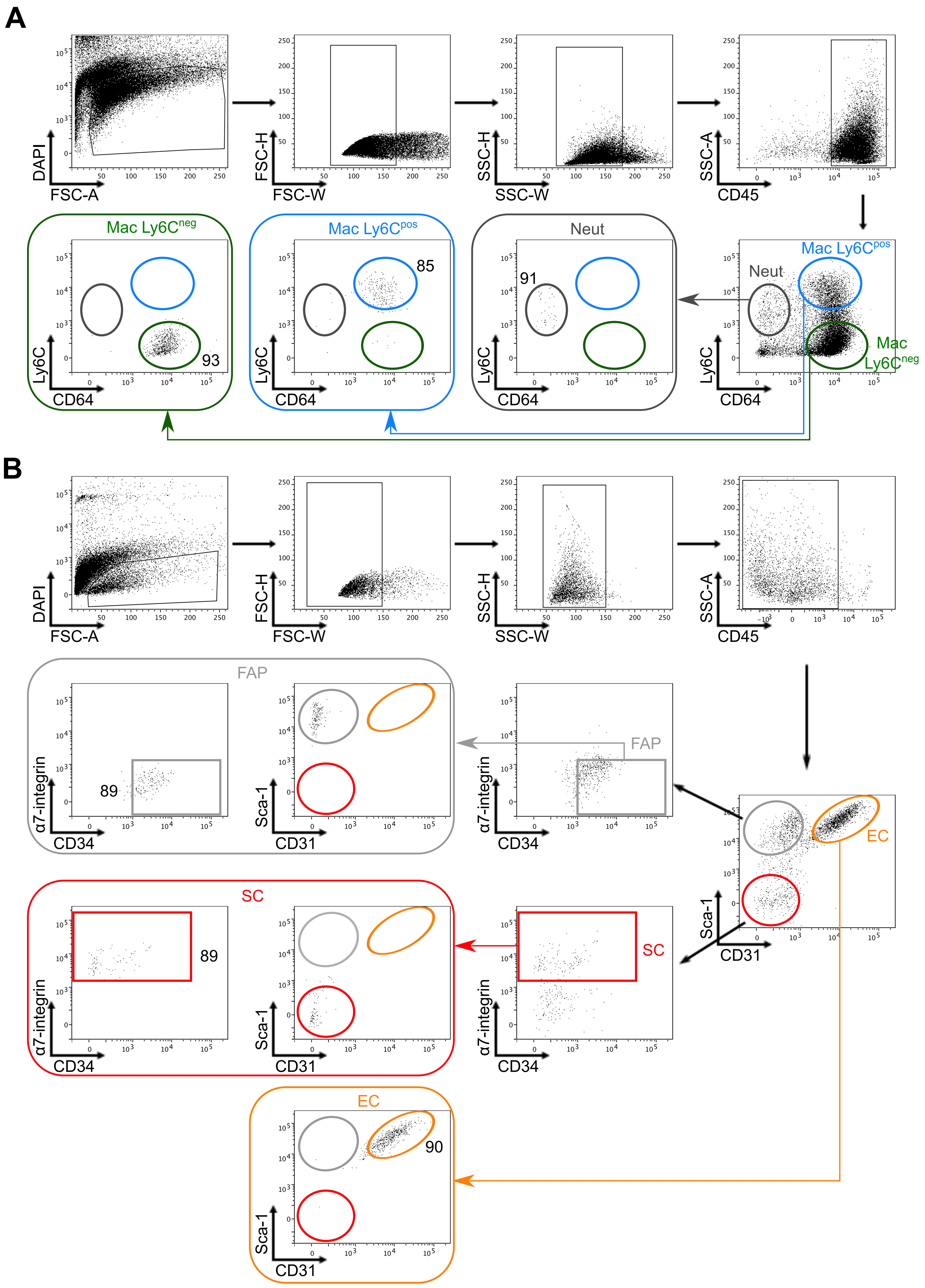

Supplementary Figure 2

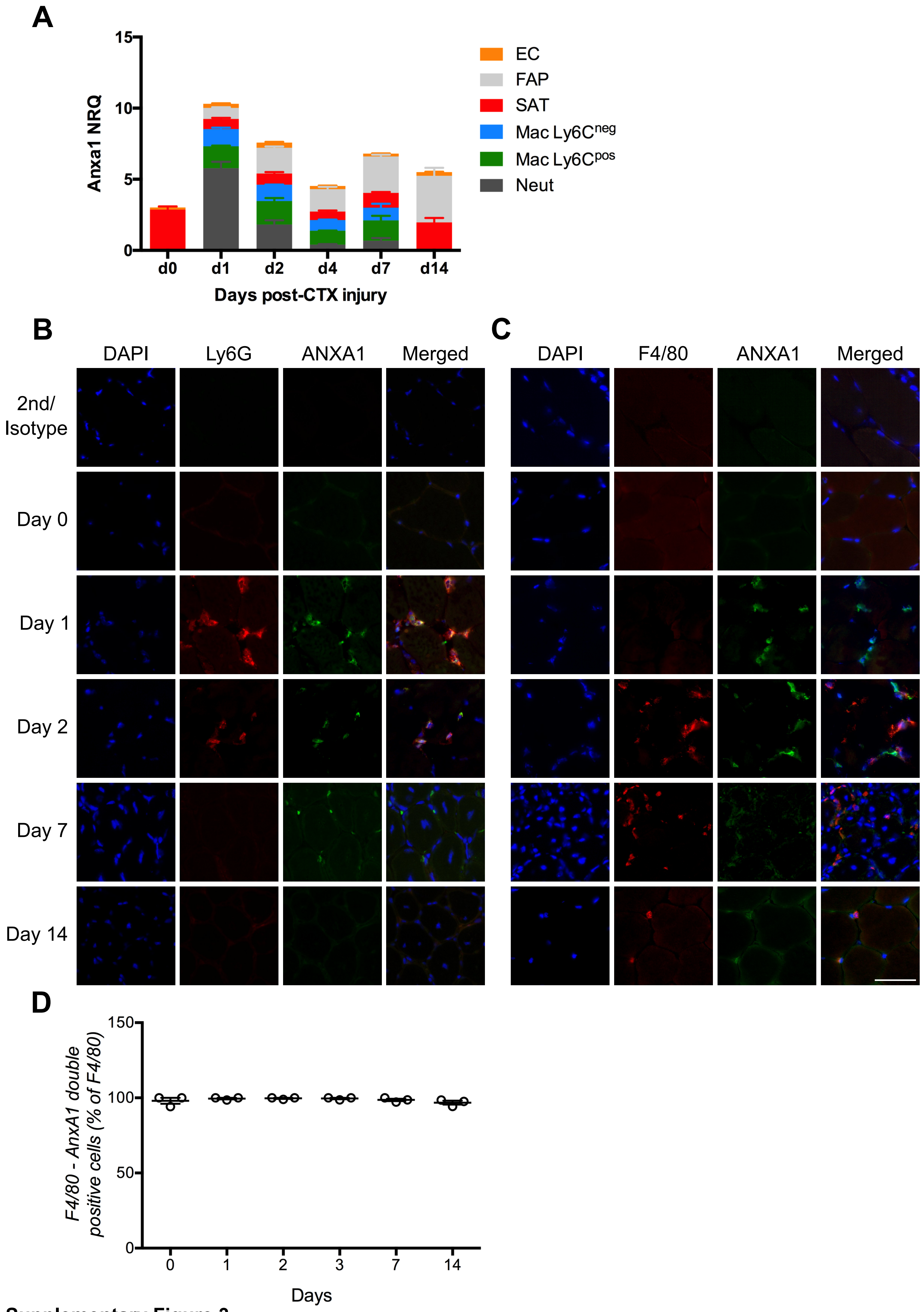

Supplementary Figure 3

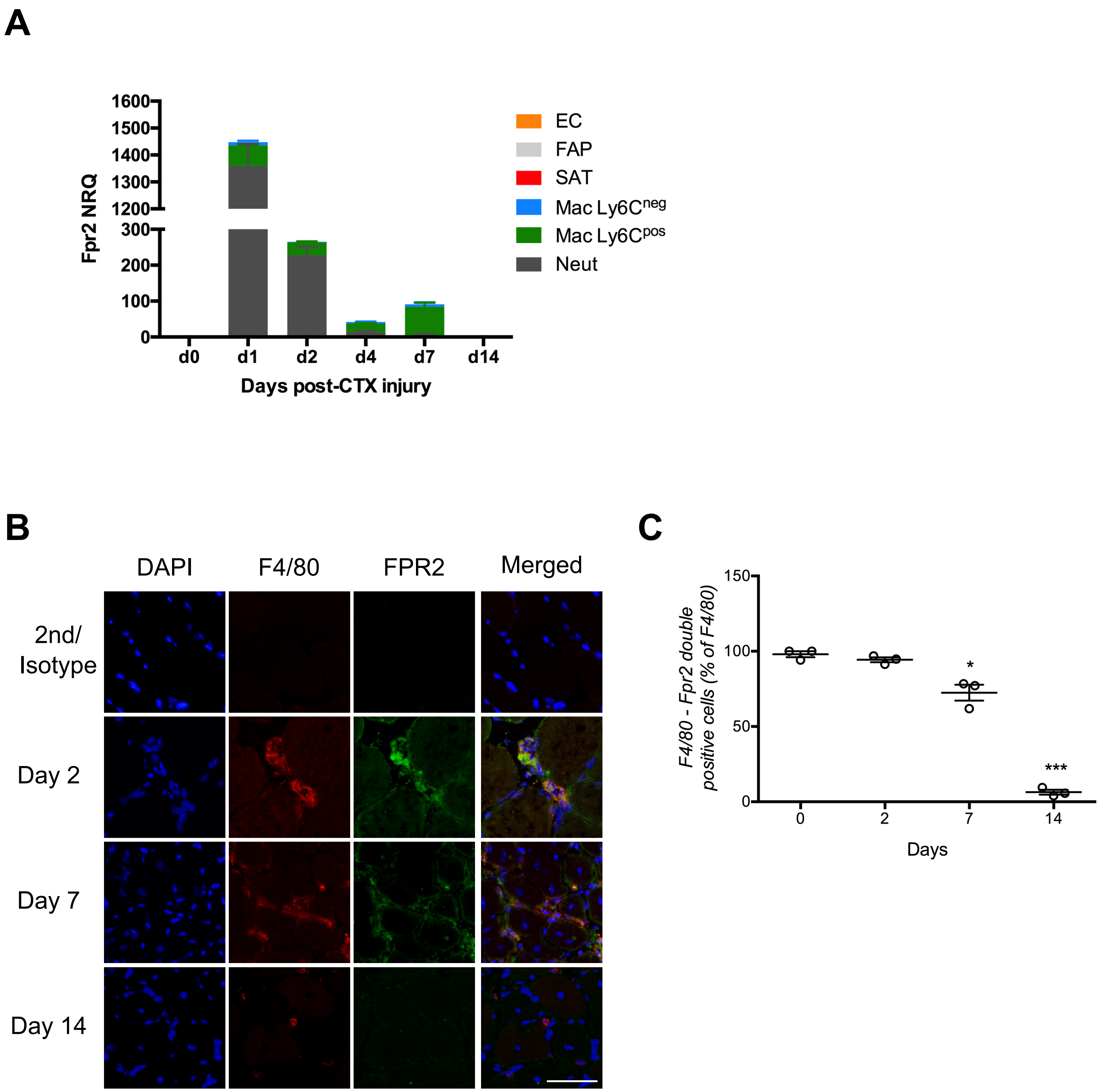

Supplementary Figure 4

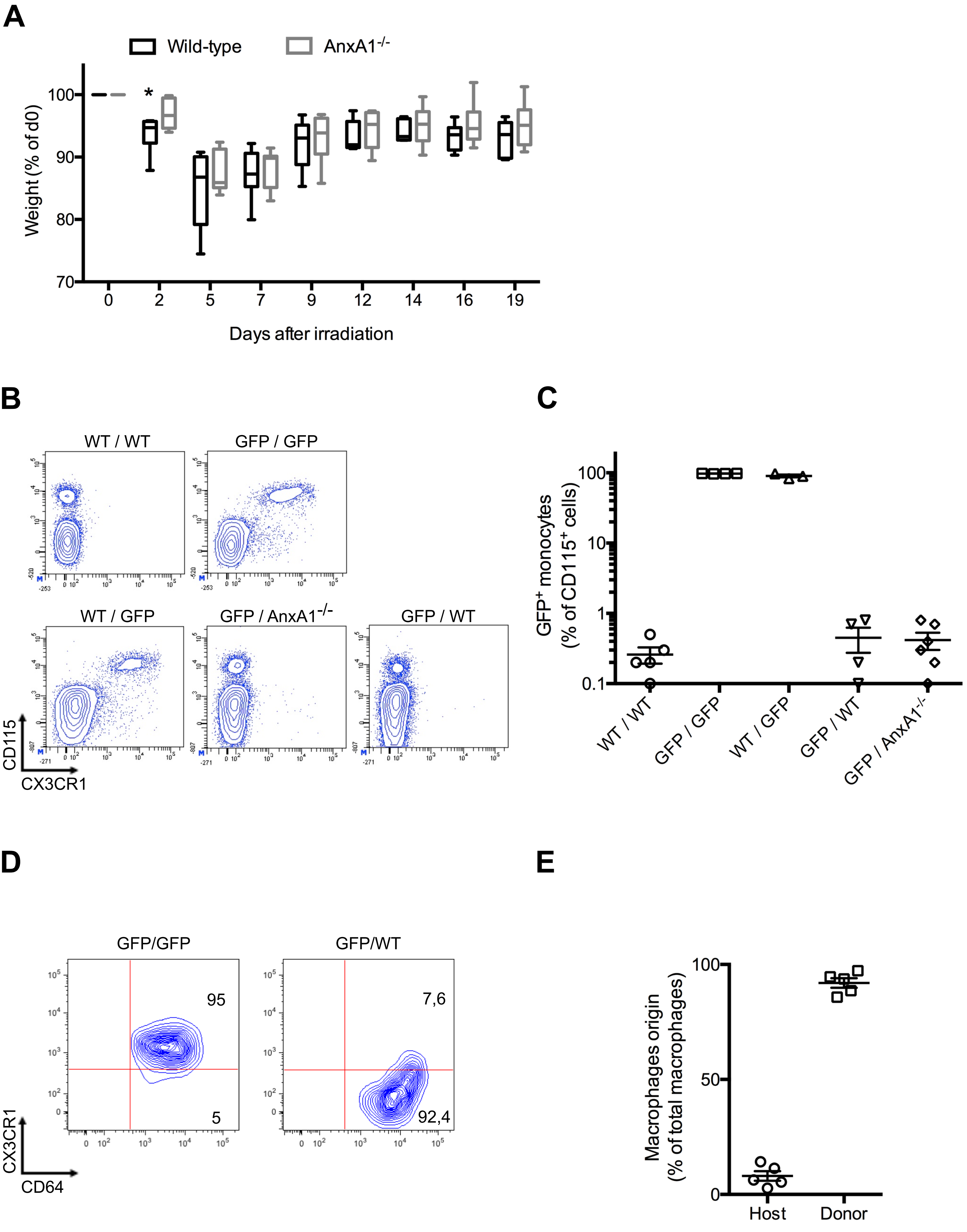

Supplementary Figure 5

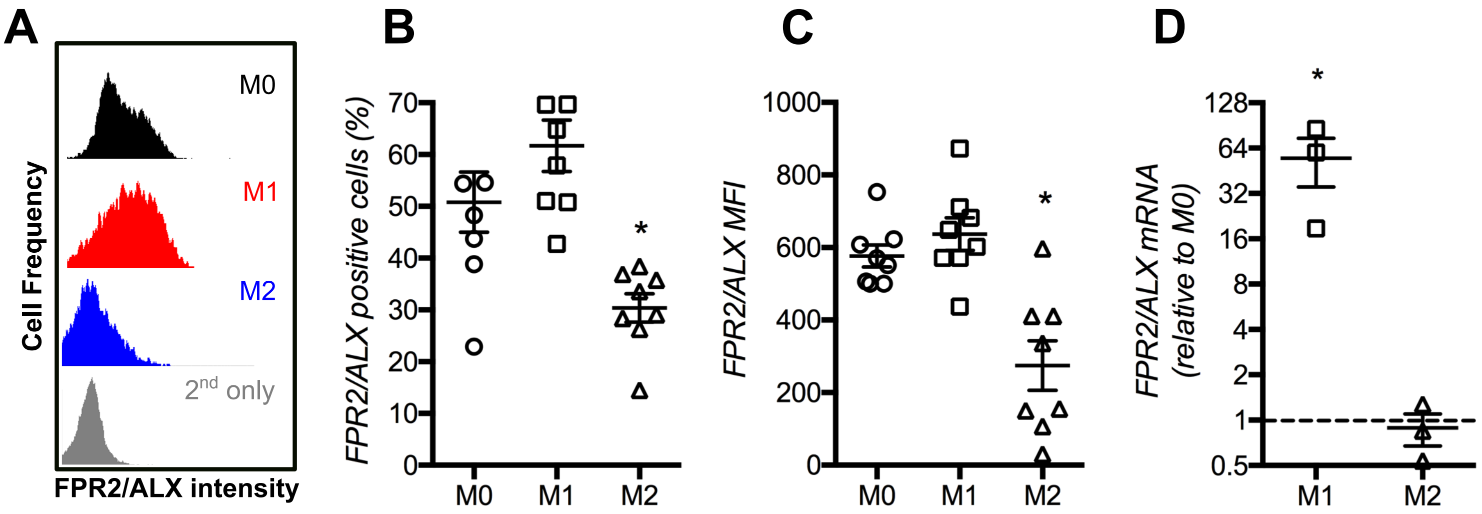

Supplementary Figure 6

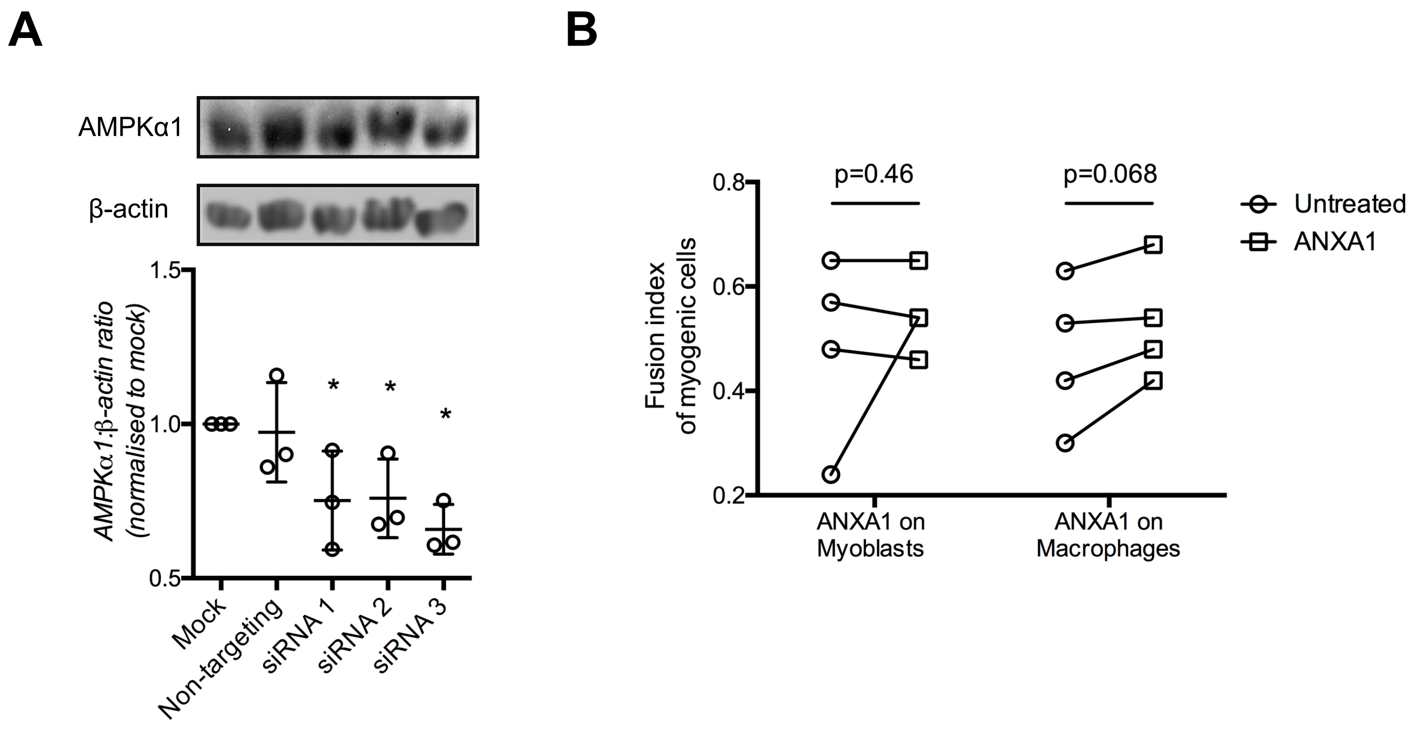

Supplementary Figure 7
